# How much does a liter of donor human milk cost? Cost analysis of operating a human milk bank in Italy

**DOI:** 10.1186/s13006-022-00530-4

**Published:** 2022-12-20

**Authors:** Guglielmo Salvatori, Domenico Umberto De Rose, Maria Clemente, Cristina Gentili, Giovanni Paride Verardi, Patrizia Amadio, Maria Paola Reposi, Pietro Bagolan, Andrea Dotta

**Affiliations:** 1 Neonatal Intensive Care Unit and Human Milk Bank, Medical and Surgical Department of Fetus - Newborn – Infant, “Bambino Gesù” Children’s Hospital IRCCS, Rome, Italy; 2 Neonatal Intensive Care Unit and Human Milk Bank, Department of Life and Reproductive Sciences, Verona Hospital, Verona, Italy; 3 Finance Control, Internal Control, “Bambino Gesù” Children’s Hospital IRCCS, Rome, Italy; 4 Neonatal Surgery Unit, Medical and Surgical Department of Fetus - Newborn – Infant, Bambino Gesù Children’s Hospital IRCCS, Rome, Italy

**Keywords:** Newborn, Human milk bank, Donor human milk

## Abstract

**Background:**

To date, 40 Human Milk Banks (HMB) have been established in Italy; however, recent cost analysis data for operating an HMB in Italy are not available in the literature.

**Methods:**

This study was a cross-sectional study performed at “Bambino Gesù” Children’s Hospital in Rome, Italy in 2019. We assessed the one-year operational costs and, the per liter unit costs at our HMB.

**Results:**

During the 2019 year we collected 771 l of human milk supplied by 128 donors. The total cost was € 178,287.00 and the average cost was € 231.00 per liter. € 188,716.00 would have been spent had the maximum capacity for 904 l been reached. We found a significant difference (€ 231.00 vs € 209.00 per liter, *p* = 0.016) comparing the cost for collected liters in the year 2019 and the cost for the maximum capacity of the bank for that year of activity. Analyzing each cost item that determines the charge of donor human milk (DHM), the highest costs are the salaries of medical and paramedical staff, and then the costs related to transporting. If the HMB works at maximum capacity and manages a greater number of liters of milk, this can represent an important saving. Conversely, the price of consumables is modest (i.e., the price of a single-use kit for breast pumps was € 0.22 per unit).

**Conclusion:**

The costs for a liter of DHM are quite high, but they must be related to the benefits, especially for preterm infants. Comparing the cost for collected liters in 2019 and the costs for the 2019 maximum capacity of the HMB, we calculated how much fixed costs of collection and distribution of DHM can be reduced, by increasing the volume of milk collected. To the best of our knowledge, this is the first complete cost analysis for an Italian Milk Bank. A thorough analysis could help to abate fixed costs and reduce the cost of a liter of DHM. The centralization of DHM can allow savings, rather than creating small HMBs scattered throughout the territory that would operate with lower milk volumes.

## Background

Breast milk (BM) and donor human milk (DHM) from the Human Milk Bank (HMB) represent the optimal nutrition for all babies, from full-term babies to preterm and high-risk newborns, due to their anti-infectious properties, immune modulators, growth hormones, and the best nutrient composition available for infants for their physiological growth [1, 2]. Several papers have correlated nutrition with human milk with a reduced incidence of respiratory and gastrointestinal infections, and re-hospitalization rates [3, 4]. The advantages of BM and, in large part, of DHM are particularly important for preterm neonates, and babies undergoing surgery. BM and DHM reduce the incidence of necrotising enterocolitis (NEC), retinopathy of prematurity, bronchopulmonary dysplasia, and some complications of surgery for congenital heart diseases, brain malformations, thoracic and gastrointestinal abnormalities or those involving other organs [5, 6]. In these more vulnerable infants, BM and DHM are better tolerated and absorbed than formula, allowing an earlier full enteral feeding, an earlier parenteral nutrition suspension, and earlier central venous catheter removal [7]. The first choice is to offer raw and unpasteurized mother’s own milk (MOM); when MOM is not available or there is not enough, DHM obtained from well-established HMBs should be used [8–10].

However, this is not an inexpensive process. Limited data are available about the cost of DHM in Neonatal Intensive Care Units (NICUs) worldwide. Carroll and Herrmann found in the US in 2013 a wide variation in the mean cost of DHM per infant, ranging from US$27.04 to US$590.90 during hospitalization (about US$133.00 per liter) [11]. Buckle and Taylor analyzed seven studies with verifiable DHM costs and 17 with verifiable NEC treatment costs: they concluded that DHM provides short-term cost savings by reducing the incidence of NEC, while the incremental length of stay associated with NEC was about 18 days for medical NEC and 50 days for surgical NEC [12]. A recent observational study quantified the cost of NEC as $46,103.00 (95% CI $16,829.00 − $75,377.00) [13].

Fengler et al. reported how providing preterm infants with DHM was significantly more expensive than using formula or MOM, but the cost of pasteurization was minimal (only € 3.51 per liter) [14].

However, a recent meta-analysis [15] showed a clear effect of any human milk in reducing NEC (all stages included), with an absolute risk reduction of 3.6% (from 1.8 to 4.8 fewer cases / 100). Therefore, cost appears to be sustainable considering improvements in morbidity of other neonatal diseases: less incidence in late-onset infections [16], bronchopulmonary dysplasia [17, 18], retinopathy of prematurity [19], and improved neurodevelopment [20]. Additionally, the use of breast milk results in a shorter length of stay [21]. Indeed, Johnson et al. reported that, after controlling for NEC in a regression analysis, each additional mL / kg / day of human milk during the first 14 days of life in preterm infants significantly decreased non-NEC − related NICU costs by US$534.00 [21].

No recent data on costs of collection and management of DHM in Italy are available: the aim of our study was to estimate the one-year operational costs and the per liter unit costs of the Bambino Gesù Children’s Hospital Human Milk Bank.

## Methods

### Study design

This study was a cross-sectional study performed at “Bambino Gesù” Children’s Hospital, Rome, Italy in 2019. The activity-based costing (ABC) method usually used for estimating the costs of blood banks was used to estimate the one-year operational costs and the per liter unit costs at our HMB [22].

This study does not contain any studies with human participants performed by any of the authors. For this type of study informed consent is not required.

For reporting our modelling study, we followed the Consolidated Health Economic Evaluation Reporting Standards (CHEERS) Statement [23].

### Setting

The “Bambino Gesù” Children’s Hospital HMB was established in 1989 and is the sole Human milk Bank in Rome and in the whole area of Lazio, connected to NICUs and part of the Italian Association of Donor Human Milk Banks. We follow and sustain breastfeeding specific advice providing support to mothers who are breastfeeding or who must maintain milk production during their baby’s stay in the NICU [24]. The production process was developed based on “Italian National Recommendations for the Organisation and Management of Human Milk Banks as a Tool for the Protection, Promotion, and Support of Breastfeeding”.

[10, 25]. DHM is pasteurized utilizing the Holder pasteurization method (62.5 °C for 30 minutes) before use, to eliminate most bacterial pathogens and viral transmission [26]. As indicated by the Italian guidelines, the use of the Hazard Analysis and Critical Control Points (HACCP) is strongly suggested [27].

Processed DHM is provided in hospitals to preterm and low birth weight infants, children with congenital heart disease, and high-risk surgical infants, according to the provider’s prescription.

### Population

Donors for our HMB are mothers whose babies are admitted to our hospital, and nursing mothers who have seen recruitment information. They are carefully selected within 12 months of lactation if they are in good general health (the most important points are not smoking, not drinking, no recreational or other drugs) and have then tested negative for Human Immunodeficiency Virus, Hepatitis B, Hepatitis C, and syphilis. In addition, a bacteriological count is carried out on a small aliquot of breast milk once per human donation, and then randomly during the donation period [28]. Donors receive no monetary compensation for the donation. They obtain milk by mechanical pump or by manual extraction for a total period of 3 months. The milk is collected in disposable containers of polypropylene and stored in the freezer compartment of the donor’s home refrigerator before delivery to the HMB. A driver from our hospital, collects the milk from donors’ homes 3 hours / day 5 days / week, using a car donated to our bank by the biggest dairy cow farm in Rome. It is important that the cold chain is never interrupted. Therefore, the milk is collected at the lactating mother’s home from her freezer; it is transported to the car in a refrigerated bag and transported by car inside a freezer that maintains the temperature at − 20 °C. Once in the hospital, the milk is brought to the HMB in a cooler bag.

The milk thus collected is then stored in a freezer at − 24 °C in the Lactarium Room. Inside the Lactarium, health workers prepare all the nutritional products of the hospital and, in particular, pasteurize the milk donated.

### Data collection and analysis

In our study, all DHM donations and consumptions are expressed in the international unit of a liter. All costs used in the ABC program are expressed in euros (€).

To facilitate calculation, we excluded costs incurred by donors using their mechanical pump and storage in their home freezer compartment, and we included only certified data collected from budgets, financial and expenditure reports of “Bambino Gesù” Children’s Hospital HMB in 2019.

We calculated direct and indirect costs of collection and management of a liter of milk to our bank’s DHM:

- HMB and Lactarium direct costs

- Clinical personnel (doctor, health workers, dietician);
- Medical supply;
- Laboratory tests;
- Transport (including fuel and car park);

b) HMB and Lactarium indirect costs

- Indirect supplies (such as computer printing paper and toner, stationery items);
- General costs (electricity, gas, water, telephone, data transmission, insurance, safety and quality certification, housekeeping, dietary, laundry, billing, human resources, information technology. ..);
- Depreciation of infrastructure.

The general costs, the staff costs and the depreciation of infrastructure allocation basis was the percentage of HMB’s personnel costs compared to the hospital’s total cost of personnel (0.04%).

Then we allocated the assigned indirect costs down to each collected liter. For the sole doctor who worked not only in the HMB but also in the Neonatal Intensive Care Unit, charges were calculated according to the working hours spent at each cost center. In order to assess direct costs of collection and transport of milk, we analysed the driver’s compensation, insurance, tires, and gasoline costs.

We estimated also direct and indirect costs of the Lactarium, analysing every single cost for the preparation and pasteurization of bottles of DHM, and then for the room cleaning.

We also evaluated the charges for liters of our DHM delivered to four other hospitals in Rome.

Results are expressed as mean ± standard deviation (SD) if not differently indicated, and the *P*-value was set at 0.05. Comparisons between groups were made using paired t-tests. Statistical analysis was carried out by using the Statistical Package for Social Sciences (SPSS Inc., Chicago, Illinois, USA), version 13.0.

## Results

We collected 771 l of human milk in 2019. Donors came from mothers whose sick babies are in our hospital (47 / 128 women, 37%) and volunteering at-home nursing mothers who have seen recruitment information (81 / 128 women, 63%). The total cost for 771 l of DHM supplied by 128 donors during the study period was € 178.287.00, and the average cost was € 231.00 per liter.

Costs for collection and management of a liter of our bank’s DHM are summarised in Table 1, describing both direct and indirect costs. We found a significant difference (€ 231.00 vs € 209.00 per liter, *p* = 0.016) when comparing costs for collected liters in the year 2019 and costs for the maximum capacity of the bank as calculated for the year 2019. We spent € 178,287.00 to collect 771 l in 2019, whereas € 188,716.00 would have been spent if the maximum capacity for 904 l had been reached. All costs are reported in each particular, hereafter.

**Table 1:** Costs for collection and management of a liter of donor human milk at our human milk bank

	Direct costs (€)						Indirect costs (€)						Total costs (euro per liter)
	Staff	Medical consumables	Laboratory tests	Lactarium	Transport	Total	Other consumables	Lactarium	General costs	Rooms	Staff	Total	
**771 l collected in 2019**	80.25	1.43	1.12	54.63	24.57	**162.0**	0.68	19.81	25.13	5.76	17.75	**69.0**	**231.0**
**904 l of milk (maximum capacity of our bank in 2019)**	68.48	1.68	0.96	54.63	20.97	**147.0**	0.58	19.81	21.45	4.92	15.15	**62.0**	**209.0**

### Direct costs

The total cost of each type of direct cost during 2019 was divided by the number of the annual total donated liters, in order to have the cost / liter value. In Table 2 we report direct costs for collection and management of a liter of our bank’s DHM (including microbiological testing procedures on milk before pasteurization). Among these, the total cost of salaries of medical and paramedical staff (€ 21,101.00 and € 40,798.00 respectively) and transport (€ 18,955.00) was fixed.

**Table 2:** Direct costs for collection and management of a liter of donor human milk at our human milk bank

	Total costs (€)	Liters collected in 2019	Cost (€ / liter)	Total costs (€)	Liters prepared at maximum capacity	Cost (€ / liter)
**Medical staff**	21,101.0	771.0	27.37	21,101.0	904.0	23.34
**Paramedical staff**	40,798.0		52.92	40,798.0		45.13
**Medical consumables**	Point estimate			Point estimate		
**Direct costs of Lactarium**	Point estimate			Point estimate		
**Laboratory tests (microbiological testing procedures on milk before pasteurization)**	865.0	771.0	1.12	1014.0	904.0	1.12
**Transport**	18,955.0		24.58	18,955.0		20.97

In Table 3 we describe the prices of medical consumables for collection and management of a liter of DHM. Even if expensive, the costs related to transporting DHM are not the highest among those that determine the charge of DHM, after the costs for medical and paramedical staff have been accounted for (Table 4): while the sole doctor works mainly in NICU (only 0.15 full-time equivalent (FTE) in the HMB), paramedical staff members involved in milk processing work full time in HMB (1.0 FTE).

**Table 3:** Direct costs of medical consumables for collection and management of a liter of donor human milk at our human milk bank

	Amount in 2019	Price per unit (€)	Total	Liters collected in 2019	Cost per liter	Amount in 2019	Price per unit (€)	Total	Liters prepared at maximum capacity of bank	Cost per liter
130 mL single-use feeding bottle	128.0	0.23	29.70	771.0	0.04	150.0	0.23	34.80	904.0	0.05
250 mL single-use feeding bottle	3900.0	0.26	1025.70		1.33	4570.0	0.26	1201.99		1.56
Single-use kit for breast pumps	144.0	0.22	31.68		0.04	168.0	0.22	36.96		0.05
Identification labels	4028.0	0.004	16.51		0.02	4720.0	0.004	19.35		0.03
**Total costs**			**1103.59**					**1293.11**

**Table 4:** Direct costs of collection and transport of donor human milk

Details of collection and transport costs	Amount in 2019	Liters collected in 2019	Cost per liter	Amount in 2019	Liters prepared at maximum capacity of bank	Cost per liter
Driver	18,284	771.0	23.71	18,284	904.0	20.23
Insurance	153.0		0.20	153.0		0.17
Tires	80.0		0.10	80.0		0.09
Petrol	438.0		0.57	438.0		0.48
**Total costs**	**18,955**		**24.58**	**18,955**		**20.97**

### Other costs

Direct and indirect costs of the Lactarium Room are reported in Table 5. Costs of consumables per 250 mL single-use feeding bottle are available in Table 6 and were negligible (about € 1.00 per 250 mL single-use feeding bottle). The time spent on different activities was used to assess costs (reported in Table 7: about 6 minutes for each bottle).

**Table 5:** Direct and indirect costs of the Lactarium

	Direct costs (€)					Indirect costs (€)					Total costs
	Staff	Medical consumables	Amortization	Maintenance	Total	Other consumables	General costs	Amortization rooms	Staff	Total	
**Feeding-bottle of pasteurized DHM**	1.59	1.02	0.11	0.02	**2.73**	0.01	0.50	0.12	0.36	**0.99**	**3.72**
**Liter of pasteurized DHM (50 mL single-use feeding bottles, (*n*** **= 20)**	31.73	20.33	2.15	0.41	**54.63**	0.30	10.08	2.31	7.12	**19.81**	**74.43**

*DHM* donor human milk

**Table 6:** Costs of consumables per 250 mL single-use feeding bottle used in the Lactarium

Consumables	Price per unit (€)	Amount	Costs (€)
250 mL single-use feeding bottle	0.27	1.0	0.27
Labels	0.004	2.0	0.008
Transparent bags 1 kg	3.22	0.1	0.32
Headcover with elastic	0.03	1.0	0.03
Disposable gloves	0.05	1.0	0.05
Non sterile gowns	0.30	1.0.	0.30
3-layer mask with elastic	0.04	1.0	0.04
750 mL disinfectant bottle	7.56	0.0002	0.001
			**1.019**

**Table 7:** Time spent for different activities in the Lactarium

Activities	Minutes for each feeding bottle	Total minutes for each activity	Median of feeding bottles per day	Minutes for each feeding bottle	Notes
**Cleaning surfaces**	**0.02**	10.0	658.0	0.02	
**Receiving / checking milk sheets**	**0.14**	90.0	658.0	0.14	
**Printing labels**	**0.30**	120.0	400.0	0.30	
**Preparing bottles**	**5.0**	5.0	1.0	5.00	
**Storage of bottles in the pasteurizer**	**0.17**	1.0	6.0	0.17	
**Registration of bottles**	**0.05**	30.0	658.0	0.05	
**Storage of bottles in the refrigerator**	**0.21**	15.0	72.0	0.21	72 feeding bottles / time
**Control of occurred pasteurization**	**0.01**	1.0	72.0	0.01	72 feeding bottles / time
**Unloading pasteurizers**	**0.21**	15.0	72.0	0.21	72 feeding bottles / time
**Preparation of thermal bags**	**0.05**	2.0	40.0	0.05	72 feeding bottles / time
**Control of pharmacy products**	**0.12**	120.0	1000.0	0.12	
**Washing grids**	**0.04**	2.0	50	0.04	
**Cleaning of pasteurizers**	**0.14**	10.0	72.0.0	0.14	
**Registration of milk batches**	**0.05**	30.0	658.0	0.05	
**Inventory**	**0.02**	15.0	658.0	0.02	
**Total minutes for sector**	**6.53**				

Starting in May 2019, our bank also started supplying DHM for free to other hospitals in our city, for a total cost of € 44,435.00: each charge is reported in Table 8.

**Table 8:** Costs of liters of our donor human milk delivered to other hospitals in Rome in 2019

Other hospitals in Rome receiving donor human milk	Liters delivered	Costs for each liter (€)	Total costs (€)
“San Giovanni Calibita –Fatebenefratelli Isola Tiberina” Hospital	**24**	**231**	**5582**
“Policlinico Umberto I” University Hospital	**33**		**7593**
“Policlinico Casilino” General Hospital	**73**		**16,780**
“Fondazione Policlinico Universitario Agostino Gemelli- IRCCS” University Hospital	**63**		**14,481**
**Total**	**192**		**44,435**

## Discussion

Breast milk represents the best food choice for all babies, especially preterm and surgical infants [29]. Whenever the mother’s own milk is unavailable or there is not enough, DHM should be used [10, 30]. There are several ways of looking at the cost-effectiveness of the use of banked DHM [31]. A systematic analysis by Buckle et al. from seven studies on the costs of DHM and 17 studies on the costs of NEC showed a positive cost-benefit ratio [12]; and a quality improvement project confirmed this finding in 55 hospitals, by analyzing how the increased availability of DHM has been associated with a decrease in the NEC rate [32].

To the best of our knowledge, our study was the first complete cost analysis for an Italian Human Milk Bank, whereas in Germany, Fengler et al. have reported an average cost of € 306.95 per liter of DHM [14]. The average cost of our DHM was lower, at about € 231.00 per liter, but higher than costs reported by Daili et al. at Shanghai Children’s Hospital (US $ 168.00 per liter) [33].

We demonstrated that the major item was the cost of staff. Therefore, the costs of DHM can be widely different in each country, according to the economic situation and to the cost of work.

We believe that the microbiological safety outweighs the minimal costs of pasteurization, that Fengler et al. estimated to be 1.1% of the cost of a liter of DHM (€ 3.51 / € 306.95) [14].

In comparing costs for collected liters in 2019 and costs for the maximum capacity of the bank, we found a significant difference (*p* = 0.016), underlining how much the fixed costs of collection and distribution of DHM can be reduced, by increasing the liters of milk collected. Therefore, this novel aspect should be carefully considered by local health organizations: the presence of several milk banks in the same region appears not justified, considering that these fixed costs would increase with more HMBs, while donor mothers from the same area can all donate milk to the same institution that manages larger volumes and distributes DHM to those who request it. Fengler’s and Daili’s groups had not addressed this aspect. Similarly, Hoodbhoy described that in the United Kingdom the estimated cost of 1 liter of donor breast milk has been estimated to be £150 – £290, but this has been not investigated [34].

The main limitations of this study include its retrospective design and the single-center site. Furthermore, we did not assess the exact cost of pasteurization per liter because we found that the time spent pasteurizing the milk was minimal. We did not include in our analysis the costs incurred by the milk donors and maternal time to donate, because we believe they are difficult to monetize and measure at home (whereas it is easier for blood donors and other donors who come to the hospital to donate). Jegier et al. previously reported that the mean cost of providing 100 mL of human milk varied from $2.60 to $6.18 for mothers who gave human milk for their very low-birthweight infants during the early NICU stay [35]. Conversely, we can speculate that donor mothers spend about 15–30 minutes expressing half a liter of milk at home. Considering a medium salary of € 1508.00 / month in Italy for about 38 hours / week [36], they would spend from about € 2.50 to € 5.00 to express half a liter of milk. However, this is not a precise estimate, whereas we carefully assessed each aspect of the collection, production, and management of DHM at our HMB.

Further multicentre studies are needed on the cost analysis of HMBs in Italy and other countries, and the economic benefits of using DHM from an HMB, as the best alternative to a MOM.

The breastfeeding rate in high-risk newborns should be increased by specific steps, sustaining mothers during NICU hospitalization and after discharge [37]. HMBs could play a key role in promoting maternal breastfeeding, health, and growth of these fragile infants and cost savings in NICUs [21]. However, human milk collection, storage, and management is not an inexpensive process. Even if the costs per liter appear high, the reduction in the incidence of NEC and of other complications related to severe prematurity likely accounts for significant savings.

Furthermore, we demonstrated that the cost of DHM can be reduced (−€ 22.00 per liter) by working at the HMB’s maximum capacity, because all fixed costs are distributed over the greater quantity of processed milk; existing staff have excess capacity to process the greater quantity without requiring additional staff; and the variable costs of consumables contribute the least to the cost per liter.

In order to provide DHM to other hospitals in the same region, it would be conceivable to create an agreement with the additional centers to share the economic burden. The pooling of resources allows for the optimization of the use of fixed costs and could potentially limit variance in variable costs.

## Conclusions

To the best of our knowledge, this is the first cost complete analysis for an Italian Human Milk Bank. A thorough analysis of collection and management of DHM, processing milk at maximum capacity of the bank and thus reducing fixed costs, could reduce the cost of a liter of DHM, and provide benefits to a larger cohort of high-risk newborns.

## Data Availability

All considered data in this study are reported in this article.

## Abbreviations

ABC: Activity-based costing
BM: Breast Milk
CHEERS: Consolidated Health Economic Evaluation Reporting Standards
DHM: Donor Human Milk
FTE: full-time equivalent
HACCP: Hazard Analysis and Critical Control Points
HMB: Human Milk Banks
MOM: mother’s own milk
NEC: Necrotising Enterocolitis
NICU: Neonatal Intensive Care Unit
